# GALD: new diagnostic tip for early diagnosis - a case report and literature review

**DOI:** 10.3389/frph.2023.1077304

**Published:** 2023-05-11

**Authors:** Silvia Zermano, Alice Novak, Emanuela Vogrig, Nadia Parisi, Lorenza Driul

**Affiliations:** ^1^ Department of Medicine, University of Udine, Udine, Italy; ^2^ Department of Obstetrics, Gynecology and Pediatrics, Department of Medical Area DAME, Obstetrics and Gynecology Unit, Udine University Hospital, Udine, Italy

**Keywords:** GALD, hydrop, immunoglobuline therapy, ultrasound, pregnancy

## Abstract

**Objective:**

Gestational alloimmune liver disease is a rare and serious condition caused by a maternal-fetal alloimmune disorder. There are not many studies about the antenatal treatment (IVIG infusion) of affected fetuses as the diagnosis is generally made postnatally. The possibility of an early diagnosis by means of ultrasonography and a gynecologist's assesment can provide prompt treatment of this disease.

**Case report:**

We report the case of 38-year-old pregnant woman referred to our centre in view of severe fetal hydrops seen by ultrasound at 31 weeks + 1 day gestation. A male infant was born and subsequently died after developing liver failure. Postmortem examination revealed the presence of diffuse hepatic fibrosis in the absence of hemosiderin deposits and no extrahepatic siderosis. Immunohistochemical analysis was also performed which showed diffuse hepatocyte positivity for the terminal complement complex (C5b-C9) confirming the suspicion of GALD.

**Methods:**

A comprehensive literature search published from 2000 to 2022 was conducted on PubMed and Scopus. Paper selection was performed following the PRISMA guidelines. Fifteen retrospective studies were identified and selected.

**Results:**

A total of 15 manuscripts describing 26 cases were finally included in our research. Twenty-two fetuses/newborns with suspected GALD were studied, of which 11 had a confirmed histopathological diagnosis of GALD. Prenatal diagnosis of gestational alloimmune liver disease is difficult because ultrasound findings may be absent or nonspecific. Only one case report described fetal hydrops similar to our clinical case. As highlighted by the current case, in fetuses presenting with hydrops, once the most common etiologies have been excluded, hepatobiliary complications and liver failure caused by GALD should be considered

**Conclusions:**

Global knowledge of this disorder and its wide spectrum of presentations may help to increase the number of cases that are diagnosed early and accurately. The recurrence rate of an infant being affected with GALD in another pregnancy is more that 90%. Recurrence however can be prevented by treatment with IVIG during pregnancy. This highlights the importance of having obstetricians and pediatricians familiar with gestational alloimmune liver disease.

## Introduction

Gestational alloimmune liver disease is a rare and serious condition caused by a maternal-fetal alloimmune disorder, which occurs antenatally and leads to severe fetal liver failure with possible neonatal hemochromatosis (1).

Several studies showed that prenatal treatment using IVIG infusion in women at risk of recurrence of gestational alloimmune disease can prevent the disease in subsequent pregnancies. However, there are not many studies about the antenatal treatment of affected fetuses as the diagnosis is generally made postnatally (2).

Therefore, the possibility of an early diagnosis by means of ultrasonography and a gynecologist's assesment can provide prompt treatment of this disease (3).

## Case presentation

A 38-year-old woman, primigravida at 31 weeks + 1 day gestation, was referred to our centre with severe fetal hydrops seen by ultrasound. The patient reported a reduction of fetal movements since the week before, on a background of an otherwise normal pregnancy. On admission in our unit, fetal ultrasound scan showed significant cephalic edema and bilateral thoracic effusion.

The patient's medical history was unremarkable. She did not report risk factors and chronic medications. There was no familial history of congenital defects.

The routine pregnancy investigations were performed and showed: positive Rhesus blood, normal blood tests, negative serological profile, normal urine tests and negative RT PCR Covid19.

The first and second trimester assessments of pregnancy were normal.

On admission the serological investigations and autoimmunity profile showed negative results. A fetal cardiotocograph trace displayed reduced variability. Spontaneous premature rupture of amnio-chorionic membranes occurred a few hours later with stained meconium. Decision for delivery by emergency cesarean section was taken.

A male infant was born with a birth weight of 2,500 g (>97°p). The 1- and 5-minute Apgar scores were 1 and 3, respectively. The pH at birth was 7,31. The infant was admitted to the intensive care unit for further observation and care. On admission he was hypotonic, unresponsive and diffusely edematous.

His laboratory tests showed thrombocytopenia, abnormal synthetic liver function (hypoalbuminemia and coagulopathy), increased liver enzymes and increased ferritin. Multiple transfusions of concentrated blood cells, platelets, and plasma were administered.

Metabolic and inherited diseases associated with early onset neonatal liver failure were ruled out. Abdominal ultrasound showed regular liver morphology and size, with smooth profile. Diffuse hyperechogenicity of parenchyma was seen with no evidence of focal lesions. Moderate intra-abdominal effusion was present in the perisplenic and perihepatic areas, as well as among the bowel loops. An echocardiogram showed no pericardial effusion and a slight increase in pulmonary pressure.

In light of a suspected diagnosis of acute liver failure (NALF), immunoglobulins IV were administered and an exanguinotransfusion was performed.

Unfortunately, the patient's condition deteriorated with the onset of early renal failure unresponsive to treatment and the appearance of multiple cerebral hemorrhagic areas documented on ultrasound and later on MRI. The patient died on the 10^th^ day of postnatal life after progressive multiple organ failure.

The postmortem exam, which was formally allowed by parents, revealed the presence of diffuse hepatic fibrosis in the absence of hemosiderin deposits and no extrahepatic siderosis. Immunohistochemical analysis was also performed and showed diffuse hepatocyte positivity for the terminal complement complex (C5b-C9).

This last analysis supports the suspected diagnosis of gestational alloimmune liver disease even in the absence of visceral hemosiderin deposits.

Genetic investigations were also performed: the karyotype was normal, Array CGH showed a duplication of maternal origin on the short arm of chromosome 10. No pathogenetic significance can currently be attributed to the latter chromosomal finding. No pathogenic variants of the Neu1 gene have been identified at WES but only of the variant of unknown significance of the NPC1 gene (c.2257G > A), which is present in heterozygotes.

## Materials and methods

A systematic review of literature was performed using Scopus (www.scopus.com, accessed on 19.07.2022) and PubMed (www.ncbi.nlm.nih.gov, accessed on 19.07.2022) databases in order to answer the clinical question. The search terms were: “GALD”, “Gestational Alloimmune Liver Disease”, “pregnancy AND GALD”, “pregnancy AND Gestational Alloimmune Liver Disease”. Articles describing one or more cases of GALD, published from January 2000 to July 2022, were identified from the above databases. Only papers written in English were included in our research. Studies were deemed eligible for inclusion in the present review if they described at least one case of GALD. All duplicate studies were excluded. Selected articles were independently reviewed by two authors (A.N. and S.Z.). The whole process is described in Table 1 and shows the list of included and excluded articles and the reason for exclusion.

**Table 1:** Prisma flow chart.

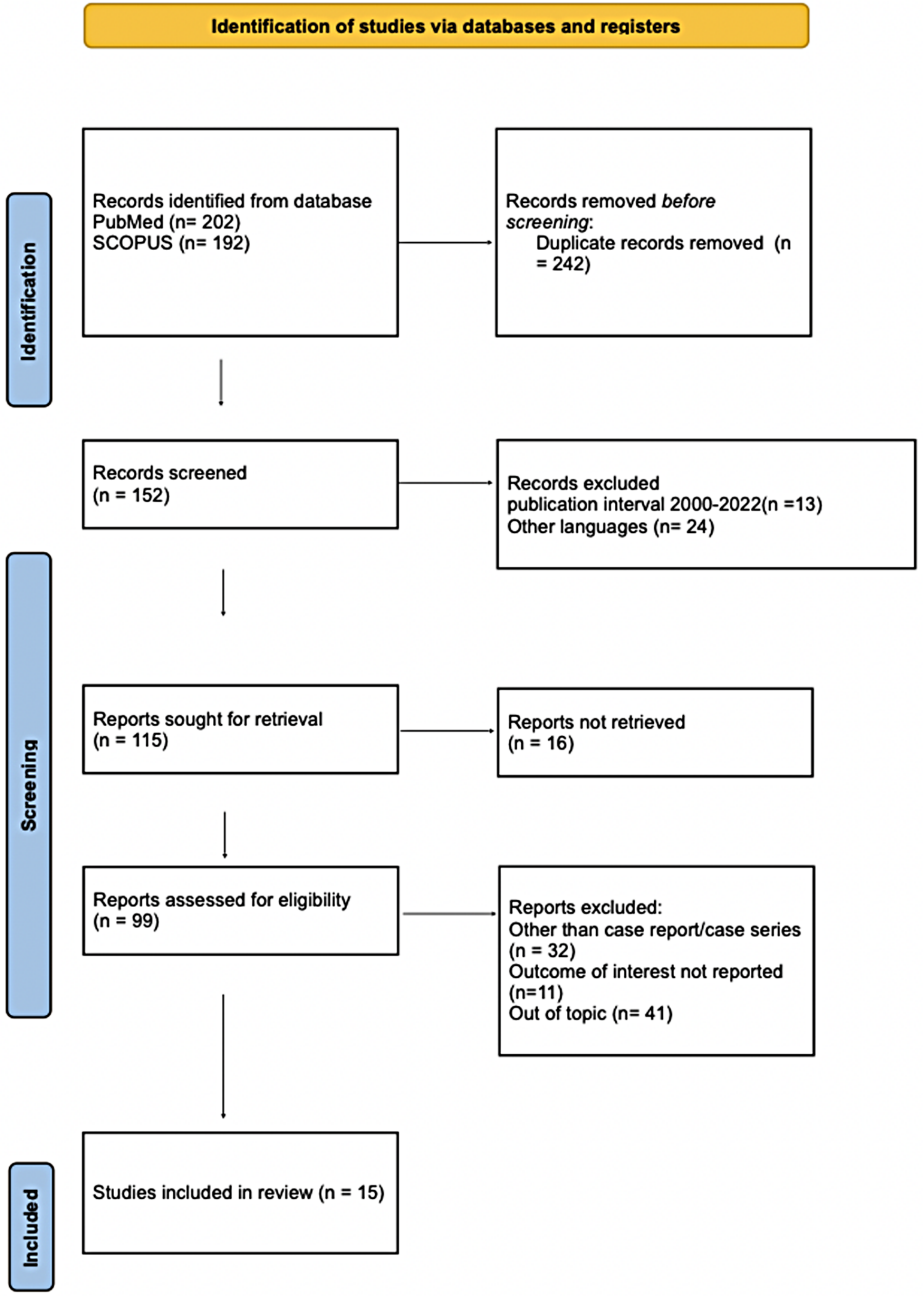

From: Page MJ, McKenzie JE, Bossuyt PM, Boutron I, Hoffmann TC, Mulrow CD, et al. The PRISMA 2020 statement: an updated guideline for reporting systematic reviews. BMJ 2021;372:n71. doi: 10.1136/bmj.n71. For more information, visit: http://www.prisma-statement.org/.

We collected the following information: maternal age, parity, gestational age, ultrasound findings, clinical presentation, fetal/neonatal outcome, treatment, microsocopic diagnosis, maternal comorbidities. All this data is listed in Table 2.

**Table 2:** Data of systematic review.

Author	Title	Maternal age	Parity	EG	Ultrasound findings	Clinical presentation	Fetal/neonatal outcome	IG Therapy during pregnancy	Histological/immunohistochemical diagnosis	Maternal comorbidities
Moorhead R.	Successful pregnancy outcomes following intravenous immunoglobulin treatment in a woman with a previous fetal death in utero due to gestational alloimmune liver disease: A case report	32	>1	34 + 2	- Fetal hydrops - Reduced fetal movements	Unknow	Fetal death	No	Yes	Low-grade squamous intraepithelial cervical lesion
Sarker M.	Intravenous immunoglobulin induced pancytopenia while preventing development of gestational alloimmune liver disease: A case report	33	0	21 + 0	- Fetal hydrop - Anhydramnios	Pulmonary hemaorrage	Neonatal death	No	No	Neonatal demise secondary to GALD
Sciard C.	Prenatal imaging features suggestive of liver gestational allo immune disease	Unknown	/	/	Case 1: - IUGR - Oligohydramnios Case 2: - IUGR - Oligohydramnios - Placental hydrops - Ascites Case 3: - IUGR - Oligohydramnios Case 4: - IUGR - Oligohydramnios - Placental hydrops - Ascites - Splenomegaly	Case 4: splenomegaly	Case 1: fetal death Case 2: neonatal death Case 3: TOP Case 4: alive	Only in one case	Yes	Unknown
Concejo Iglesias P.	Usefulness of dual gradient-echo MR imaging for the prenatal	34	0	27 + 2	- Fetal hydrops - Irregular nodular hepatic surface	Unknown	Fetal death	Yes	Yes	No
Mariano da Rocha C.R.	Neonatal Liver Failure and Congenital Cirrhosis due to Gestational Alloimmune Liver Disease: A Case Report and Literature Review	Unknown	/	31 + 4	- IUGR - Oligohydramnios	- Hypoglycemia - Cholestatic jaundice - Ascites - Hepatomegaly - Lower gastrointestinal bleeding	Neonatal death	No	Yes	No
Casas-Alba D.	Broadening the spectrum of neonatal hemochromatosis	30	/	39 + 6	/	- Metabolic acidosis - Liver failure - Hepatic siderosis - Extra hepatic siderosis	Alive	Yes (not in pregnancy)	No	No
Yeh P.J.	Efficacy of Intravenous Immunoglobulin/Exchange Transfusion Therapy on Gestational Alloimmune Liver Disease	Unknown	0	37 + 3	/	Twin A: - Ascites - Splenomegaly - High ferritin Twin B: Normal	Alive	Yes (not in pregnancy)	Yes	ANA+Anti-DNA+↑ IgG level ↓ C3 level
Anastasio H.B.	Gestational Alloimmune Liver Disease.A Devastating Condition Preventable With Maternal Intravenous Immunoglobulin	30	0	29	- IUGR - Oligohydramnios	- Hepatic failure - Coagulopathy - Multi organ failure - Splenomegaly - Pulmonary congestion	Neonatal death	No	No	No
Tsunoda T.	Neonatal liver failure owing to gestational alloimmune liver disease without iron overload	26	0	34	- IUGR	- Low birth weight - Cholestasis - High serum ferritin	Alive (liver transplantation)	Yes (not in pregnancy)	Yes	No
Clarke NE.	Fulminant liver failure in a neonate	25	0	27 + 6	- IUGR - Echogenic bowel - Echogenic kidneys	- Liver dysfunction - Renal impairment	Neonatal death	No	Yes	No
Midorikawa H.	Disparate clinical findings in monochorionic twins with neonatal hemochromatosis	Unknown	/	36	Twin A: Oligohydramnios Twin B: Normal	Twin A: - Liver failure - High serum ferritin Twin B: Normal	Alive	Yes only for twin A (not in pregnancy)	No	No
Flores-Torres J.	PIGA Mutations Can Mimic Neonatal Hemochromatosis	22	0	37	- IUGR - Oligohydramnios - Pericardial effusion	- Low birth weight - Hyperbilirubinemia - Pericardial effusion - Dismorfic facial features (PIGA mutation at WES)	Alive	Yes (not in pregnancy)	No	No
Hutchings G.	Plasmapheresis as an Alternative to High-Dose Intravenous Immunoglobulin in the Prevention of Gestational Alloimmune Liver Disease	27	>1	/	1st pregnancy: - IUGR i 2nd pregnancy: terminated (diagnosis of sickle cell) 3rd pregnancy: - IUGR - fetal death at 36 w 4th pregnancy: normal 5th pregnancy: -SGA - reduced fetal movements 6th pregnancy: -SGA	1st:liver failure (liver transplantation) 5th: -Hepatic failure 6th: -Transaminitis - High serum ferritin	1st: neonatal death 2nd: terminated 3th: fetal death 4th: alive 5th: neonatal death 6th: alive	Only 6th case	Yes (only 1stcase)	Sicke cell trait
Iskandar A.T.P.	Neonatal hemochromatosis attributed to gestational alloimmune liver disease treated with IVIG and exchange transfusion therapy: an evidence-based case report	29	0	33	- Oligohydramios - Suspicion of parvovirus infection	- Encephalopathy - Poor liver function	Alive	Yes (not in pregnancy) + plasmapheresis	No	No
Mc Adams R.M.	Ileal atresia and multiple jejunal perforations in a premature neonate with gestational alloimmune liver disease	/	/	32 + 3	- Oligohydramnios - Reduced fetal movements - Ascites - Bowel obstruction - Adrenal mass	- Ileal atresia with multiple jejunal perforation, - CID, - Laboratory test suggestive for GALD	Alive	Yes (not in pregnancy)	No	No

Twelve articles were not included because they did not report the outcome of interest and did not report any information about the pregnancy.

The main risk of bias of this work is that all papers selected in the literature are case reports and case series.

We use the Preferred Reporting Items for Systematic Reviews and Meta-Analyses (PRISMA) guidelines to elaborate this review and the literature quality has been evaluated through the use of the CARE (Case Report) check list.

Due to the rarity of this occurrence, we presented data in a descriptive manner.

## Results

We described our clinical case and then we performed a literature review with MEDLINE (PubMed) and Scopus. We analyzed 15 manuscripts describing 26 cases. A summary of the characteristics is presented in Table 2.

Twenty-two fetuses/newborns with suspected GALD were studied. Of these, 11 had a histopathological diagnosis of GALD. In the other cases GALD diagnosis was made based on suggestive clinical findings and imaging assessments. The mean maternal age is 28,8 years; however, this information is reported in only 10 cases.

Gestational age at presentation ranges from a minimum of 21 + 0 weeks to a maximum of 39 + 6 gestational weeks. Six women were nulliparous. Only three women had comorbidities; one woman was a carrier of sickle cell trait (4), another had a previous diagnosis of LSIL (5) while the third presented with an elevated serum immunoglobulin G level, a low complement level, positive antinuclear antibody and positive anti-double-strand DNA antibody (6). However, none of these comorbidities have been described as an association with the development of GALD in pregnancy.

The clinical presentation is extremely heterogeneous. It was only in three case reports that the patient reported reduced fetal movement, as was in our case.

Only ten infants with diagnosis of GALD survived. Eight of them underwent post-natal intra-venous immunoglobulin therapy (IVIG), One case had IVIG combined with plasmapheresis (8), while two babies underwent a liver transplant (4, 9).

Regarding the ultrasound findings, the review showed that the most representative parameter was fetal growth restriction which was present in 13 fetuses (59%) followed by oligohydramnios present in 10 out of 22 fetuses (45%); fetal ascites (6/22, 27%) and pericardial effusion (3/22, 14%). Placental edema (3/22, 14%) was rare. Only one case described fetal hydrops as was the presentation in our case, but this case developed additional ultrasonographic signs such as severe placental edema, inferior vena cava dilatation and an irregular and nodular liver surface (10); A single case reported the following: splenomegaly, echogenic bowel and echogenic kidneys (11).

## Discussion

Gestational alloimmune liver disease (GALD) is a maternal-fetal alloimmune disorder involving the fetal liver, often resulting in neonatal liver failure (12, 13).

The damage starts antenatally when the fetus receives the maternal IgG, between the 17th and 22nd week of gestation. This period has been suggested to be the time of liver injury onset in affected fetuses (14, 15).

GALD can present anytime from 18 weeks gestation to 3 months post-delivery (16).

The incidence of GALD seems to be very rare on the basis of the cases reported in literature.

So far, There is no gold diagnostic tool for GALD. Despite the most common presentation benign neonatal hemochromatosis, extrahepatic siderosis is not found in up to 40% of cases of gestational alloimmune hepatopathy, as it was revealed in our case, demonstrating the spectrum of disease phenotypes (1).

Immunohistochemical staining for C5b-9 complex, the neoantigen created by the action of complement, is pathognomonic for gestational alloimmune liver disease (17).

However, if the condition is not suspected, immunostaining may not be performed and the diagnosis can be easily missed.

Detecting liver failure due to GALD can be challenging, especially because no serologic tests are available. Consequently, the diagnosis of GALD is made postnatally. Suspicion for this complication in pregnancy should be raised if there's a maternal history of multiple fetal deaths or a previous sibling with neonatal liver failure (7).

Prenatal diagnosis of gestational alloimmune liver disease is difficult because ultrasound findings may be absent or nonspecific. There is usually a history of intrauterine growth restriction, oligohydramnios and prematurity (18).

As highlighted by the current case, in fetuses presenting with hydrops, once the most common etiologies have been excluded, hepatobiliary complications and liver failure caused by GALD should be taken into account (19–23).

## Conclusions

Global knowledge of this disorder and its wide spectrum of presentations may help to increase the number of cases that are diagnosed in a timely manner.

In addition, once a woman has delivered an infant with GALD, the probability that the next pregnancy will be affected is greater than 90%. Recurrence can however be prevented by treatment with IVIG during pregnancy (1, 24).

It is very important for obstetricians and pediatricians to be familiar with gestational alloimmune liver disease.

## Data Availability

The original contributions presented in the study are included in the article, further inquiries can be directed to the corresponding author.
